# Supplementary material for: Data linkage to evaluate the long-term risk of HIV infection in individuals seeking post-exposure prophylaxis
Source: Nat Commun. 2021 Feb 22;12:1219. doi: 10.1038/s41467-021-21485-w (PMC7900236; doi:10.1038/s41467-021-21485-w)
Supplement: Supplementary file 1 — Supplementary Information [file 41467_2021_21485_MOESM1_ESM.pdf]

SUPPLEMENTARY INFORMATION

Data linkage to evaluate the long-term risk of HIV infection in individuals seeking post-exposure prophylaxis

Frédérique Hovaguimian, Huldrych F. Günthard, Christoph Hauser, Anna Conen, Enos Bernasconi, Alexandra Calmy, Matthias Cavassini, Marco Seneghini, Alex Marzel, Henriette Heinrich, Alexandra Scherrer, Julien Riou, Adrian Spoerri, Kurt Schmidlin, Suraj Balakrishna, Dominique L. Braun, Silvana K. Rampini, Jan S. Fehr, Roger D. Kouyos and the Swiss HIV Cohort Study

Supplementary table 1. HIV incidence rates in MSM seeking PEP at the University Hospital of Zurich

Population	Nb of person-years	Nb of events	HIV incidence rates (per 10'000 person-years)
MSM seeking PEP	3119.9	22	70.5
MSM seeking PEP, excluding possible PEP failures	3109.1	16	51.5
MSM with repetitive PEP seeking	493.2	4	81.1
MSM with repetitive PEP seeking, excluding possible PEP failures	491.9	3	61

MSM: men who have sex with men; PEP: post-exposure prophylaxis.

Supplementary figure 1

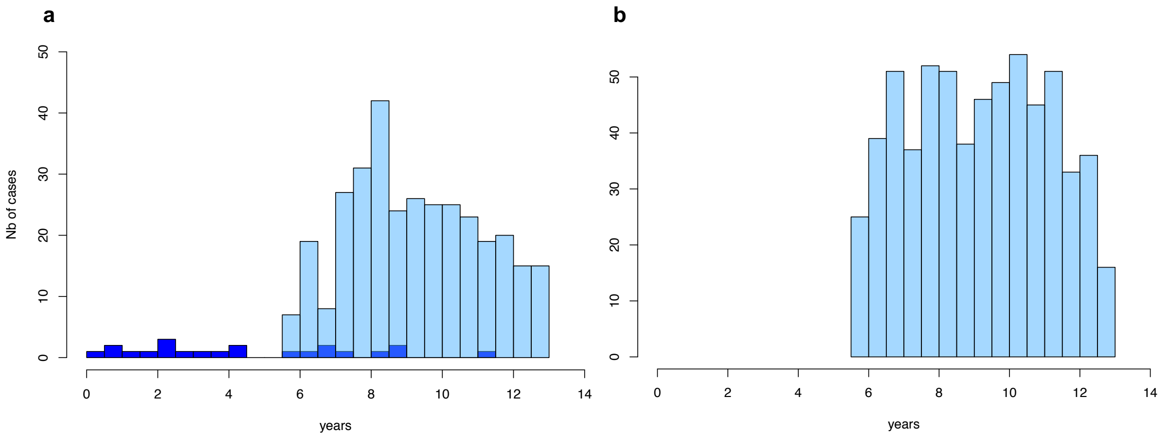

Supplementary figure 1 Distribution of time-at-risk among MSM (a) and non-MSM (b) PEP seekers. Histogram illustrating that the distribution of follow-up times in MSM was similar to non-MSM PEP seekers. Dark blue bars indicate individuals diagnosed with HIV. MSM: men who have sex with men; PEP: post-exposure prophylaxis.

Supplementary note 1: list of data routinely collected by the attending physician during PEP consultation, as reported by Marzel et al.[1]

> Demographic data (age, sex, nationality)
> Characterization of the event leading to post-exposure prophylaxis consultation, including type of sexual intercourse [i.e. insertive, receptive, versatile (insertive and receptive), anal, vaginal, oral, smear of body fluids on healthy or wounded skin or mucous membranes, hand/feet to genitals contact, condom use, condom dysfunction], hours since exposure, or additional risk factors for HIV transmission (i.e. menstruation, ejaculation, and sexually transmitted infections)
> Result from the HIV screening test at presentation

SUPPLEMENTARY REFERENCES

1 Marzel, A. *et al.* Prescription of Postexposure Prophylaxis for HIV-1 in the Emergency Room: Correct Transmission Risk Assessment Remains Challenging. *J Acquir Immune Defic Syndr* **74**, 359-366, doi:10.1097/QAI.0000000000001265 (2017).
