# Supplementary material for: Data linkage to evaluate the long-term risk of HIV infection in individuals seeking post-exposure prophylaxis
Source: Nat Commun. 2021 Feb 22;12:1219. doi: 10.1038/s41467-021-21485-w (PMC7900236; doi:10.1038/s41467-021-21485-w)
Supplement: Supplementary file 3 — Reporting Summary [file 41467_2021_21485_MOESM3_ESM.pdf]

nature research

Corresponding author(s): Frédérique Hovaguimian

Last updated by author(s): Jan 21, 2021

Reporting Summary

Nature Research wishes to improve the reproducibility of the work that we publish. This form provides structure for consistency and transparency in reporting. For further information on Nature Research policies, see our Editorial Policies and the Editorial Policy Checklist.

Statistics

For all statistical analyses, confirm that the following items are present in the figure legend, table legend, main text, or Methods section.

n/a	Confirmed	
☐	☒	The exact sample size (n) for each experimental group/condition, given as a discrete number and unit of measurement
☒	☐	A statement on whether measurements were taken from distinct samples or whether the same sample was measured repeatedly
☐	☒	The statistical test(s) used AND whether they are one- or two-sided<br>*Only common tests should be described solely by name; describe more complex techniques in the Methods section.*
☐	☒	A description of all covariates tested
☒	☐	A description of any assumptions or corrections, such as tests of normality and adjustment for multiple comparisons
☐	☒	A full description of the statistical parameters including central tendency (e.g. means) or other basic estimates (e.g. regression coefficient) AND variation (e.g. standard deviation) or associated estimates of uncertainty (e.g. confidence intervals)
☐	☒	For null hypothesis testing, the test statistic (e.g. F, t, r) with confidence intervals, effect sizes, degrees of freedom and P value noted<br>*Give P values as exact values whenever suitable.*
☒	☐	For Bayesian analysis, information on the choice of priors and Markov chain Monte Carlo settings
☒	☐	For hierarchical and complex designs, identification of the appropriate level for tests and full reporting of outcomes
☒	☐	Estimates of effect sizes (e.g. Cohen's d, Pearson's r), indicating how they were calculated

Our web collection on statistics for biologists contains articles on many of the points above.

Software and code

Policy information about availability of computer code

Data collection	Data constituting the PEP-USZ database were collected using Microsoft Access (2013, version 15.0); Data constituting the Zurich Primary HIV study (ZPHI) and Swiss HIV Cohort Study (SHCS) databases were collected using Microsoft Access (2016, version 16.0) and Oracle (version 12.2), respectively.
Data analysis	All statistical analyses were conducted in R, version 3.6.1.

For manuscripts utilizing custom algorithms or software that are central to the research but not yet described in published literature, software must be made available to editors and reviewers. We strongly encourage code deposition in a community repository (e.g. GitHub). See the Nature Research guidelines for submitting code & software for further information.

Data

Policy information about availability of data

All manuscripts must include a data availability statement. This statement should provide the following information, where applicable:

- Accession codes, unique identifiers, or web links for publicly available datasets
- A list of figures that have associated raw data
- A description of any restrictions on data availability

Datasets analyzed during the current study and used to generate table 1, figure 3 and 4, and supplementary information are not publicly available due to the sensitive nature of the data yielded by this small, highly representative, individual-level dataset (see also: http://www.shcs.ch/294-open-data-statement-shcs). Source data are thus not provided with this paper. Investigators with a request for selected data should send a proposal to the Swiss HIV Cohort Study (SHCS) address (www.shcs.ch/contact). The provision of data will be considered by the Scientific Board of the SHCS and the relevant study team. Data provision is subject to Swiss legal and ethical regulations, and will be detailed in a material and data transfer agreement.

Field-specific reporting

Please select the one below that is the best fit for your research. If you are not sure, read the appropriate sections before making your selection.

[x] Life sciences [ ] Behavioural & social sciences [ ] Ecological, evolutionary & environmental sciences

For a reference copy of the document with all sections, see nature.com/documents/nr-reporting-summary-flat.pdf

Life sciences study design

All studies must disclose on these points even when the disclosure is negative.

Sample size	We did not perform sample size calculations, as the size of the PEP seekers cohort was defined by the fixed size of the available PEP-USZ database (975 records collected over 6 years). Additionally, the main aim of this study was descriptive, i.e. hypothesis testing so as to obtain effect estimates related to a specific intervention/exposure was not the primary aim of this study.
Data exclusions	Individuals seeking post-exposure prophylaxis for non-consensual sex or those with a positive HIV-test at hospital admission were not considered.
Replication	This item is not relevant to our study, as no experiments were conducted (data-linkage study). A certain degree of reproducibility was ensured by the fact that all internals links (i.e. sharing a common unique identifier) were also identified by external linkage, which used a different methodological approach (privacy-preserving probabilistic record linkage).
Randomization	This item is not relevant to our study, as no intervention was conducted (data-linkage study) and the study design was retrospective.
Blinding	Blinding of patients (PEP seekers) and outcome assessors (those making the HIV diagnosis) is not relevant in the context of this retrospective data-linkage study: there was no prospective follow-up of PEP seekers by outcome assessors, as PEP seekers were not enrolled in a cohort per se; outcome assessors (those making the HIV diagnosis) were independent health care providers with no role in the current data-linkage study. The external linkage used a third, independent party to conduct probabilistic record linkage after all relevant data were encrypted. This third party did not have access to the results of the internal linkage.

Reporting for specific materials, systems and methods

We require information from authors about some types of materials, experimental systems and methods used in many studies. Here, indicate whether each material, system or method listed is relevant to your study. If you are not sure if a list item applies to your research, read the appropriate section before selecting a response.

Materials & experimental systems

n/a	Involved in the study	
[x]	[ ]	Antibodies
[x]	[ ]	Eukaryotic cell lines
[x]	[ ]	Palaeontology and archaeology
[x]	[ ]	Animals and other organisms
[ ]	[x]	Human research participants
[x]	[ ]	Clinical data
[x]	[ ]	Dual use research of concern

Methods

n/a	Involved in the study	
[x]	[ ]	ChIP-seq
[x]	[ ]	Flow cytometry
[x]	[ ]	MRI-based neuroimaging

Human research participants

Policy information about studies involving human research participants

Population characteristics	All HIV-negative individuals who sought non-occupational PEP prescription between 2007 and 2013 at the University Hospital of Zurich (USZ) were considered eligible. Individuals seeking PEP for non-consensual sex or those with a positive HIV-test at hospital admission were not considered. The study population included 971 individuals, median age 32 (IQR 26 - 38), of which 797 (82.1%) were males.
Recruitment	This item is not entirely relevant in the context of this retrospective data-linkage study. The PEP-USZ database consists of routinely collected clinical data from individuals who sought PEP at the USZ between 2007 and 2013. The other 2 databases used in this project are ongoing observational cohorts: the ZPHI is a monocentric cohort study established in 2002 at the USZ, which follows longitudinally individuals aged ≥18 years with a documented primary HIV infection; the SHCS is a prospective, multicentric cohort study enrolling HIV-infected individuals aged ≥18 years in Switzerland since 1988.
Ethics oversight	Cantonal ethical committee, Zurich, Switzerland

Note that full information on the approval of the study protocol must also be provided in the manuscript.
